# Many-Faced Imitator: A Case of Acinar Adenocarcinoma of the Lung Resembling Miliary Tuberculosis

**DOI:** 10.7759/cureus.98362

**Published:** 2025-12-03

**Authors:** Ma D Valdes Bracamontes, Gangacharan Dubey

**Affiliations:** 1 Pulmonary and Critical Care, SUNY Downstate Health and Sciences University, Brooklyn, USA; 2 Pulmonary and Critical Care Medicine, South Brooklyn Hospital, Brooklyn, USA

**Keywords:** acinar adenocarcinoma, adenocarcinoma lung, ct imaging suggestive of tuberculosis, differential diagnosis of miliary tuberculosis, great imitator

## Abstract

The Great Imitator is a term used to describe different conditions that resemble other diseases. These include conditions such as syphilis, tuberculosis, lupus, systemic mycoses, and sarcoidosis, just to name some of which have fallen into this category of diseases that present with multi-system involvement, and a myriad of signs and symptoms that can be mistaken for other pathologic processes.

A high index of clinical suspicion is necessary to analyze the information obtained from history of present illness, physical exam, and information obtained from available tests (i.e., serology, microbiology, imaging). Additionally, physicians have to gather information from other sources, like family history, social history, epidemiology, risk factors associated with an individual's characteristics, such as ethnicity, occupational exposures, travel history, sick contacts, etc. To make this process more cumbersome, there is always the possibility of obtaining a false positive or false negative result from the tests we rely on to support a clinical diagnosis. Consequently, this leads to a missed diagnosis, with a delay in treatment, disease progression, and the need for follow-up studies.

Here, we discuss a case that presented with a clinical picture suspicious for tuberculosis. The patient belonged to a high-risk population to present this condition; radiologic imaging revealed a pattern consistent with miliary tuberculosis. Miliary opacities are defined as innumerable 1-4 mm pulmonary nodules scattered throughout the lung fields. Differential for miliary pattern is wide, including tuberculosis, fungal infection, and some neoplastic metastatic disease. In our case anti-tuberculosis regimen was initiated after microbiology results, but a torpid clinical course after initiation of these drugs led to performing more invasive interventions, which allowed us to obtain the diagnosis of adenocarcinoma of the lung.

## Introduction

The approach to a clinical case starts by obtaining the history of present illness and past medical history, along with additional information from social or family background that contributes to our clinical assessment. This, in association with a thorough physical exam. Additionally, the role of radiographic studies as a diagnostic aid in the decision-making process to treat chest pathology is of primordial importance. Specifically, the use of computed tomography (CT) provides more detailed information about the lung architecture. By means of pattern recognition and identification of the structures involved in a pathologic process, a diversity of differential diagnoses can be ruled in or discarded, which makes the CT a great ally when the clinical picture is rather vague, and the range of possible etiologies is extensive [1-4]. Here we present a case of a patient with suspected pulmonary tuberculosis. He is from an ethnic group with a high incidence and prevalence of tuberculosis; his clinical picture and imaging studies [5-7] were suspicious for tuberculosis. Microbiology initially reported acid-fast bacilli on sputum smear, yet histopathologic diagnosis confirmed adenocarcinoma [8-10].

## Case presentation

**Figure 1:**
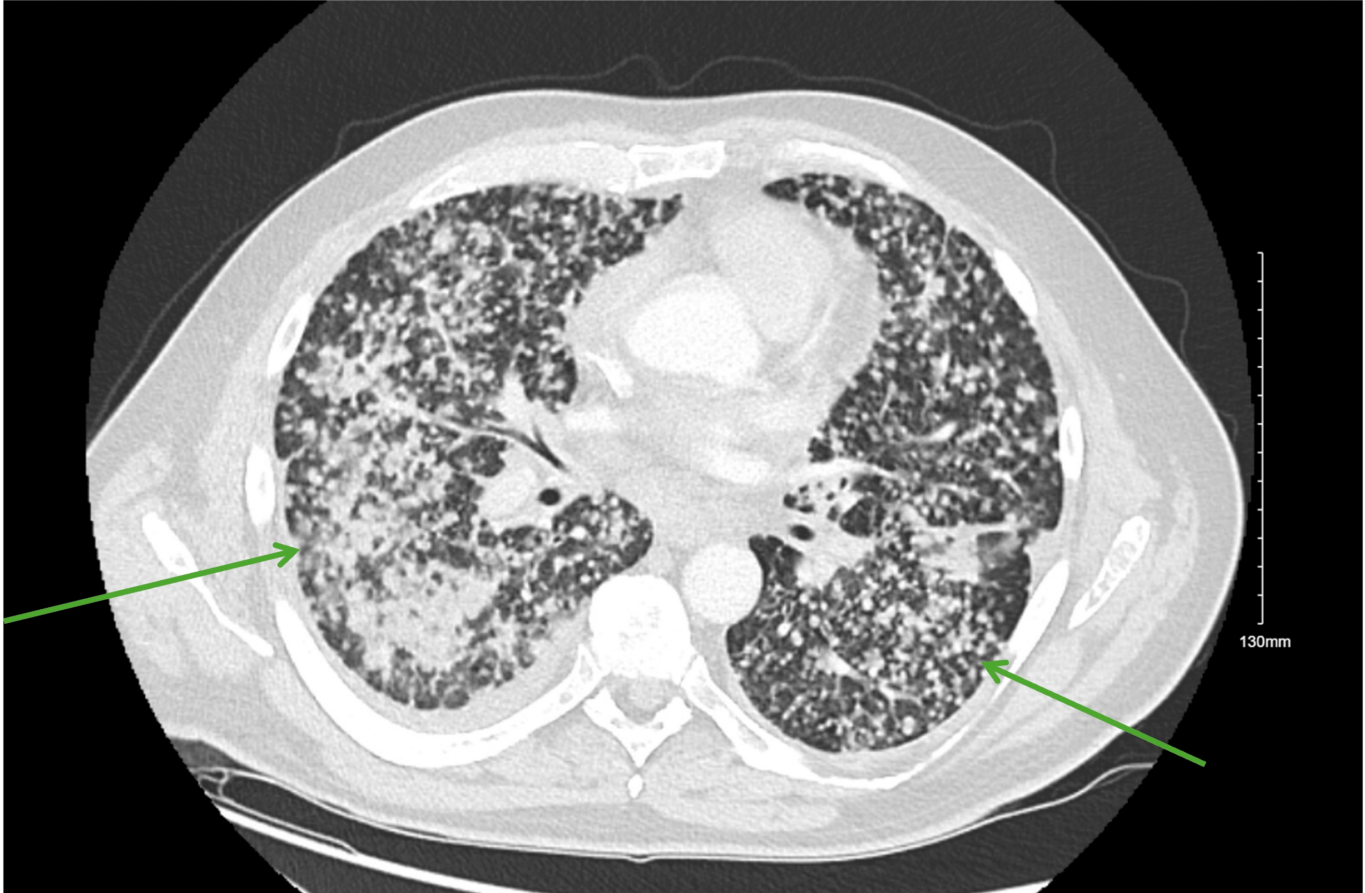
Innumerable pulmonary nodules in a miliary pattern throughout bilateral lung fields (green arrows).

**Figure 2:**
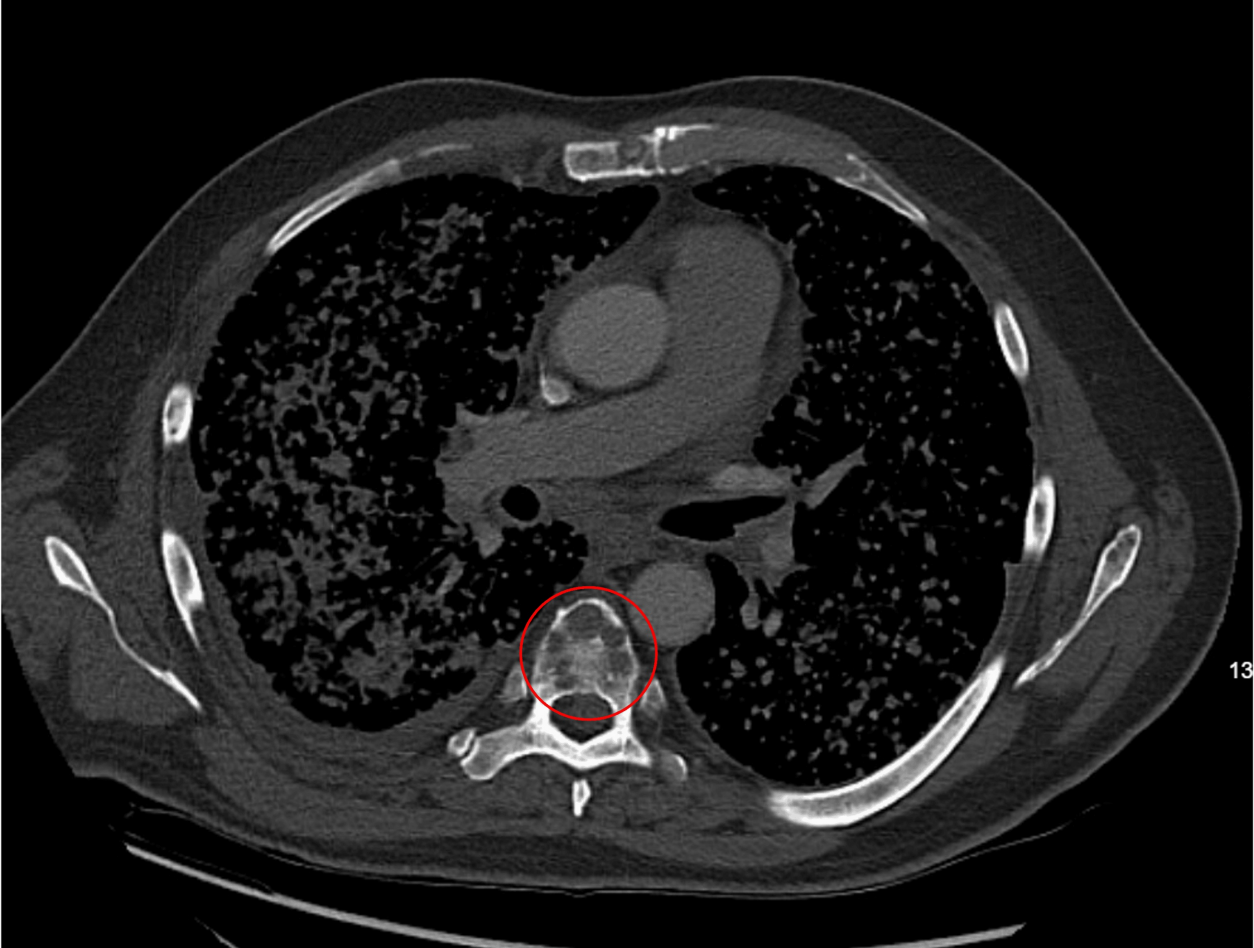
Lytic lesions in the vertebral body (red circle).

**Figure 3:**
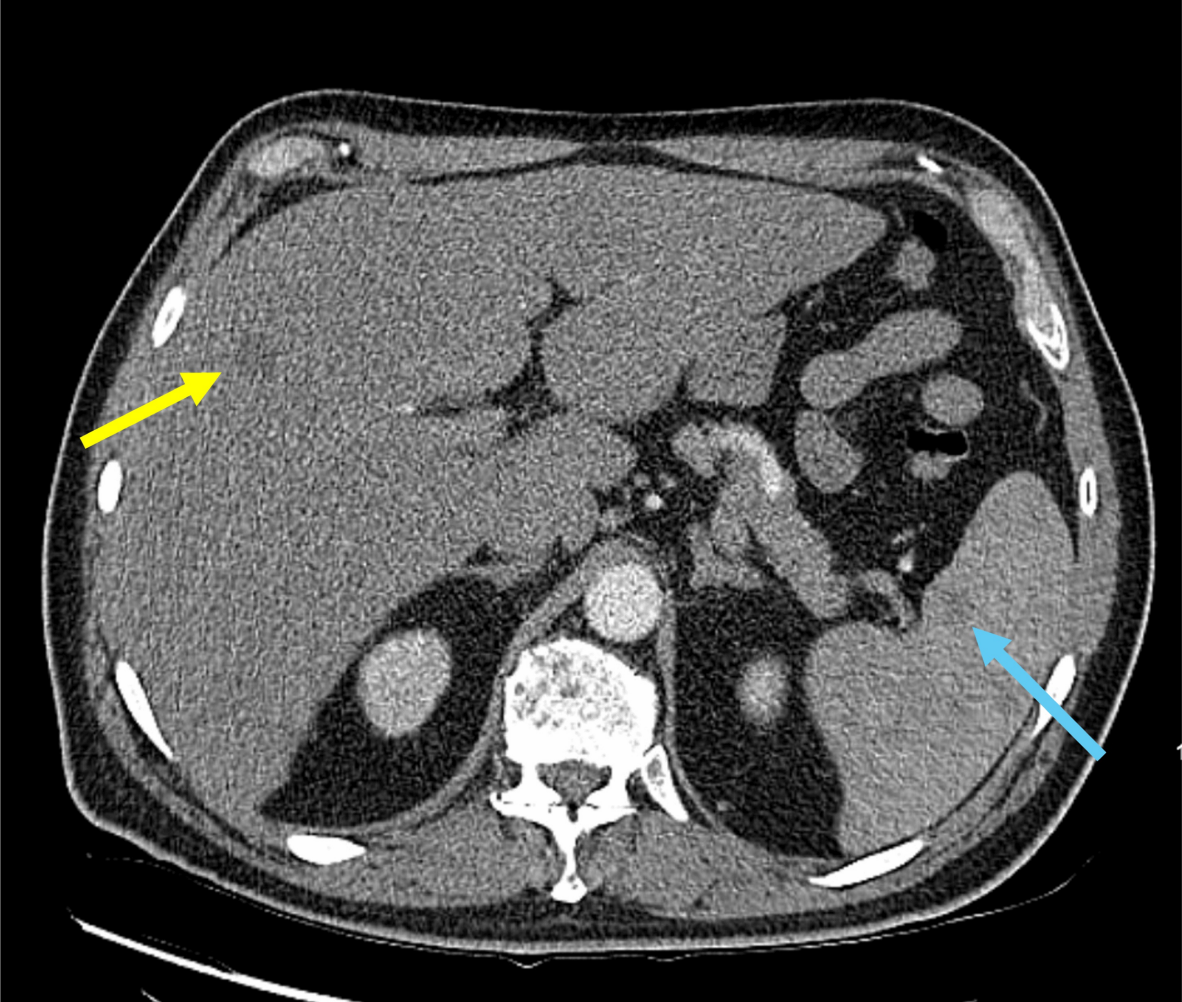
Low-attenuation lesions in the liver (yellow arrow) and spleen (blue arrow).

**Figure 4:**
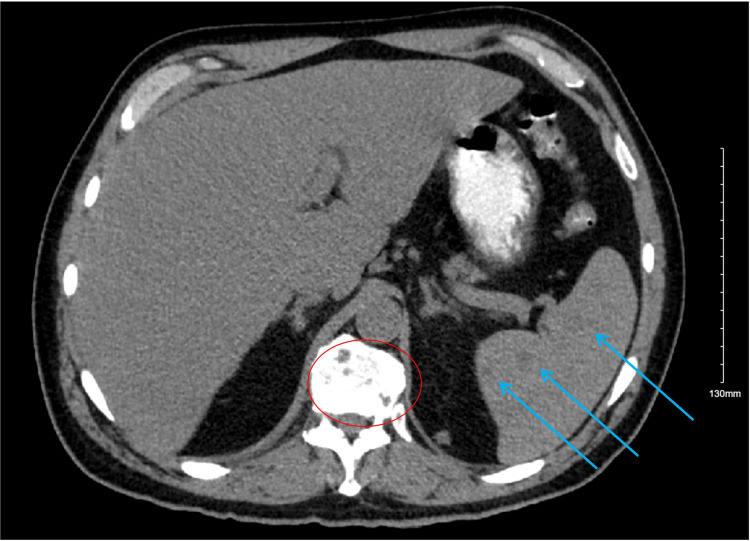
Low-attenuation lesions in the spleen (blue arrows) and lytic lesions in the vertebral body (red circle).

A 53-year-old, Hispanic/Latino male was referred to the Emergency Room by his pulmonologist to rule out tuberculosis, after a CT chest demonstrated diffuse bilateral miliary pulmonary nodules, along with osseous lesions involving the thoracic spine, as well as lesions in the liver and spleen (Figures 1-4). The patient's comorbid conditions included diabetes mellitus, hypertension, and hyperlipidemia. His clinical presentation was a productive cough for a month, with occasional streaks of blood; there were no associated fever and chills, but he had a 30 lb. weight loss, which he reported as intentional. He had no sick contacts or recent travels; his migratory status is not specified. He is a former smoker who quit 20 years ago. And reported following up with a primary care physician every year. Initial imaging results consisted of innumerable bilateral pulmonary nodules, slightly prominent on the right, many of which were coalescent. Differential diagnosis by imaging included tuberculosis, metastases, and pneumoconiosis.

**Figure 5:**
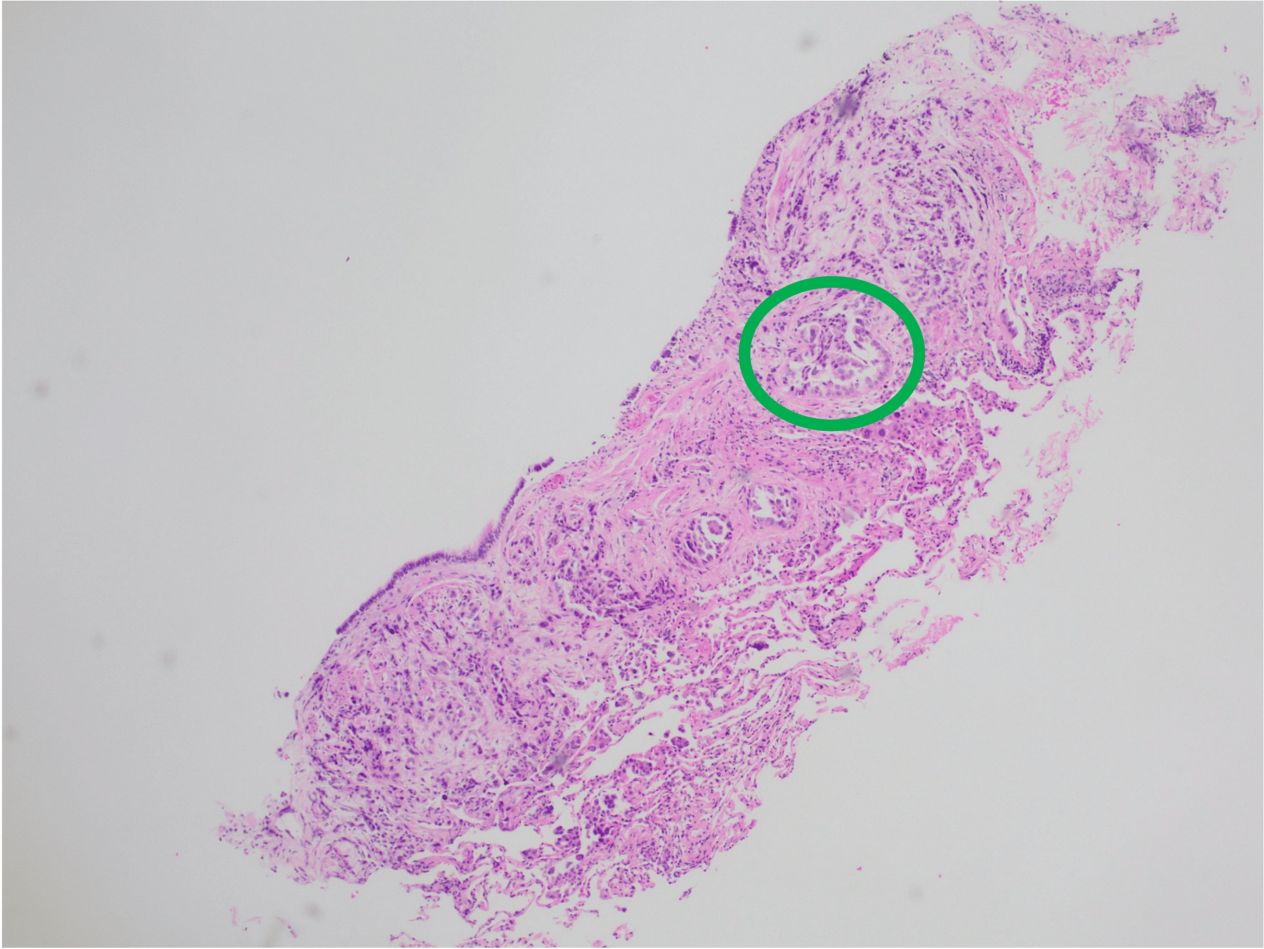
Lung tissue: transbronchial biopsy showing invasive acinar-type adenocarcinoma (green circle).

**Figure 6:**
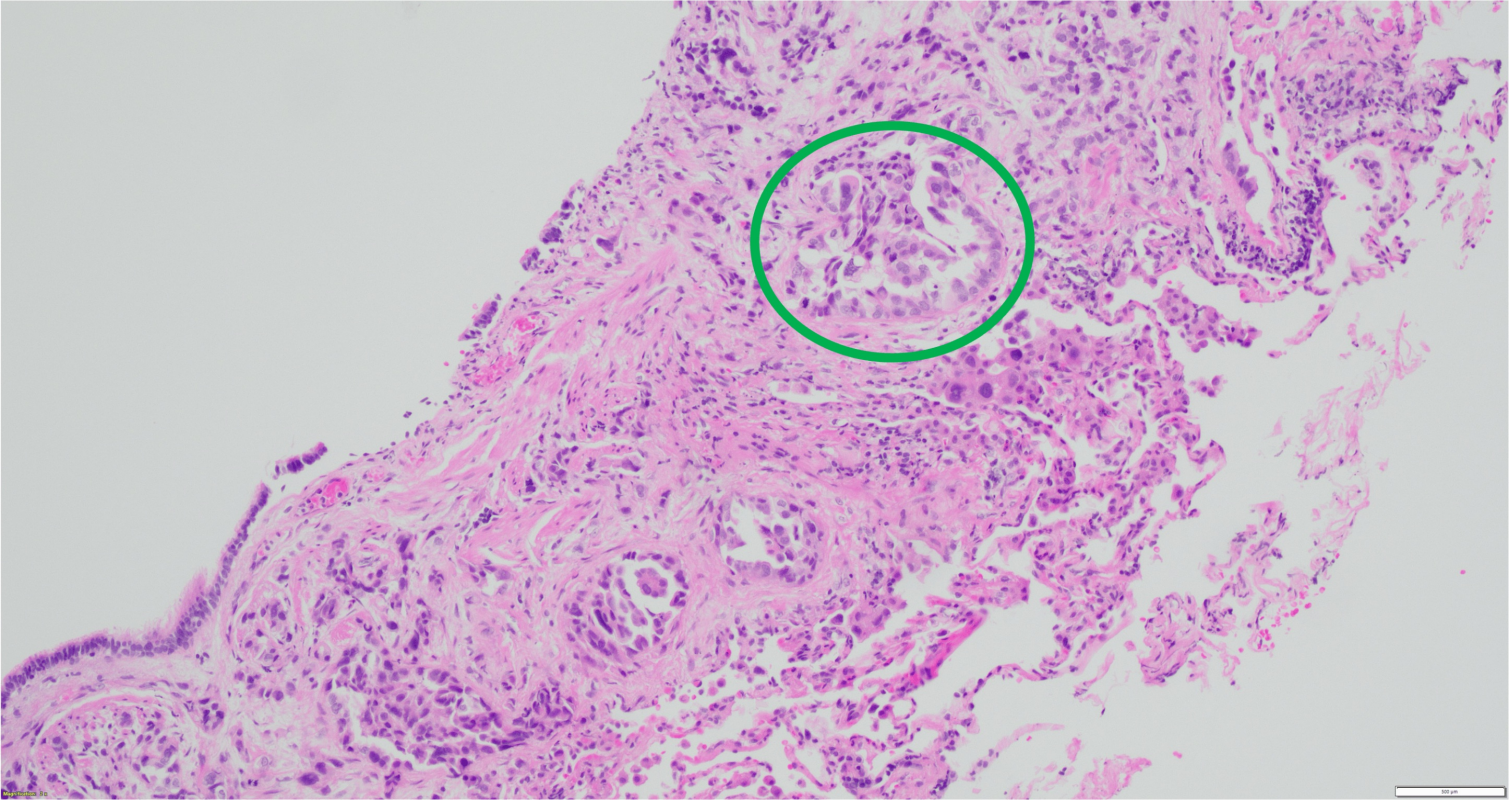
Lung, transbronchial biopsy: glandular formation characteristic of the acinar pattern (green circle).

**Figure 7:**
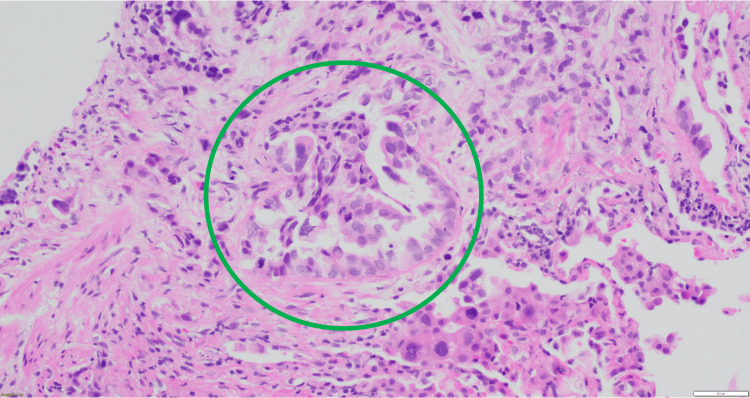
Lung, transbronchial biopsy: glandular formation characteristic of the acinar pattern. The glands are irregularly shaped (green circle).

The patient was admitted with a working diagnosis of miliary tuberculosis versus metastatic neoplasia. The QuantiFERON result was indeterminate. The first sputum sample was positive for acid-fast bacilli (AFB) on smear, but the Mycobacterium tuberculosis polymerase chain reaction (MTB-PCR) was negative. Two subsequent sputum smear tests were also negative. The patient was started on an empiric anti-tuberculosis regimen after the initial AFB smear; the regimen included isoniazid, ethambutol, rifampin, and levofloxacin, the latter chosen due to elevated transaminase levels: aspartate aminotransferase (AST) 103 U/L, alanine aminotransferase (ALT) 62 U/L. AFB sputum culture was in process and later grew normal respiratory flora and rare Haemophilus parainfluenzae. The clinical course was complicated by hypoxic respiratory failure, with oxygen requirements met by a nasal cannula and a non-rebreather mask. The patient was evaluated by Oncology, Pulmonary, and Interventional Radiology services. Since a neoplastic underlying condition was in the differential diagnosis, the patient was scheduled to undergo an imaging-guided liver biopsy, but the procedure was deferred due to increasing oxygen requirements necessitating mechanical ventilatory support, followed by a fiberoptic bronchoscopy. Upon inspection of the tracheobronchial segment, anatomy was identified and reported as normal, with no visualization of endobronchial lesions. Bronchoalveolar lavage (BAL) was performed with three saline aliquots in the right middle lobe with adequate return. Transbronchial biopsy of the right lower lobe was performed under fluoroscopy, with minimal bleeding. Cold saline was used to achieve hemostasis. Intermittent periods of desaturation were documented during the procedure, with drops to a level of 85%, which improved with adequate ventilation. No complications were reported. A transbronchial biopsy specimen was sent to pathology. Bronchial wash was sent for cytology, and BAL was sent for bacterial, fungal, and AFB cultures. The patient was then transferred to the medical intensive care unit (MICU), where he was extubated after the procedure and placed on a high-flow nasal cannula (HFNC), and subsequently, non-invasive positive pressure ventilation was started due to increased work of breathing. By day three after bronchoscopy, the work of breathing and oxygen demand led to hypoxic respiratory failure requiring invasive mechanical ventilatory support. This was followed by an episode of sinus bradycardia, and shortly after, asystole. Cardiac arrest protocol was initiated, with no return of spontaneous circulation. Bronchoscopy test results were negative for microbiology, bronchial wash cytology reported malignant cells favoring non-small cell carcinoma of the lung, and transbronchial biopsies were consistent with invasive adenocarcinoma of the acinar type (Figures 5-7).

## Discussion

Chest pathology can be challenging to diagnose based on the clinical picture; many conditions have an overlap of symptoms, and additional tests are required to establish a diagnosis. In the previous case, it was the miliary pattern identified on CT imaging that prompted the patient’s doctor to refer him for further workup. A miliary pattern is characterized by the presence of multiple sub-centimeter pulmonary nodules, usually about 3 mm in size, they are scattered between both lung fields. After this pattern is identified, the diagnostic possibilities that come to mind include infections such as tuberculosis or fungal, infiltrative processes like sarcoidosis, pneumoconiosis, or metastatic disease [1]. Regardless of the CT contribution to the diagnostic process, this is not a definitive test. Imaging data must be analyzed in the clinical context for an individual patient, along with confirmatory tests. There are reports in the literature describing lung cancer with a miliary pattern, resembling that of tuberculosis [1-4]. What made our case more challenging was that initial microbiology tests delivered a false positive result reporting the presence of AFB, prompting initiation of anti-tuberculosis medication. Although the initial plan included imaging-guided biopsy after oncology, pulmonary, and interventional radiology services' initial evaluation, the rapid clinical deterioration precluded this procedure. It was later in the clinical course of disease, after progression and worsening of symptoms, that additional tests ultimately established the diagnosis of a neoplastic malignant process [11-15].

## Conclusions

Differential diagnoses for miliary opacities include infectious and non-infectious conditions. A CT chest reporting a miliary pattern should be considered nonspecific. The clinical history is a key component in the diagnosis assessment when a patient presents from an endemic area where certain infections are prevalent. Once infectious etiologies have been ruled out, further investigative workup to rule out malignancies and metastasis should be pursued. 

Several chest diseases manifest as a set of non-specific symptoms, which frequently overlap, presenting a vague clinical scenario, requiring imaging studies and lab tests to establish a diagnosis. Furthermore, clinicians face an additional challenge when radiologic images or laboratory tests have confounding results. Additionally, anti-tuberculous drugs should not be initiated before accurate confirmation of tuberculosis diagnosis and other differential diagnoses have been ruled out. This may be followed by other tests or invasive procedures. Thus, a pathologic diagnosis should be pursued in the appropriate context to establish an accurate diagnosis, and a multidisciplinary approach should be taken to deliver the standard of care and accomplish the best outcomes in patient care.
